# The Twofold Role of 12-Hydroxyoctadecanoic Acid (12-HOA) in a Ternary Water—Surfactant—12-HOA System: Gelator and Co-Surfactant

**DOI:** 10.3390/gels4030078

**Published:** 2018-09-12

**Authors:** Katja Steck, Claudia Schmidt, Cosima Stubenrauch

**Affiliations:** 1 Institute of Physical Chemistry, University of Stuttgart, Pfaffenwaldring 55, 70569 Stuttgart, Germany; katja.steck@ipc.uni-stuttgart.de; 2 Department of Chemistry, University of Paderborn, Warburger Straße 100, 33098 Paderborn, Germany; claudia.schmidt@uni-paderborn.de

**Keywords:** gelled complex fluids, gelator, lyotropic liquid crystals, phase diagram

## Abstract

Gelled lyotropic liquid crystals can be formed by adding a gelator to a mixture of surfactant and solvent. If the gel network and the liquid-crystalline phase coexist without influencing each other, the self-assembly is called orthogonal. In this study, the influence of the organogelator 12-hydroxyoctadecanoic acid (12-HOA) on the lamellar and hexagonal liquid crystalline phases of the binary system H_2_O–C_12_E_7_ (heptaethylene glycol monododecyl ether) is investigated. More precisely, we added 12-HOA at mass fractions from 0.015 to 0.05 and studied the resulting phase diagram of the system H_2_O–C_12_E_7_ by visual observation of birefringence and by ^2^H NMR spectroscopy. In addition, the dynamic shear moduli of the samples were measured in order to examine their gel character. The results show that 12-HOA is partly acting as co-surfactant, manifested by the destabilization of the hexagonal phase and the stabilization of the lamellar phase. The higher the total surfactant concentration, the more 12-HOA is incorporated in the surfactant layer. Accordingly, its gelation capacity is substantially reduced in the surfactant solution compared to the system 12-HOA–*n*-decane, and large amounts of gelator are required for gels to form, especially in the lamellar phase.

## 1. Introduction

Gelled complex fluids [1], in which the mechanical stability of a gel is combined with the microstructure of a complex fluid, are interesting candidates for (trans-)dermal drug delivery systems and tissue healing applications [1,2]. Additional applications of this class of materials can be found in a recent review by Stubenrauch and Gießelmann [1]. Gelled complex fluids can be obtained by either adding a gelator to a complex fluid or by replacing the solvent of a gel by a complex fluid. A special class of gelled complex fluids are gelled lyotropic liquid crystals (gelled LCs), such as gelled lamellar phases L_α_ (gelled L_α_). Examples of gelled L_α_ phases_,_ known in literature are the cell [3], lamellar biogels [4], and gelled lamellar phases of synthetic surfactants. Only two systems of the latter type have been studied to date: The gelled lamellar phase of the system H_2_O–*n*-decane/12-hydroxyoctadecanoic acid (12-HOA)—tetraethylene glycol monodecyl ether (C_10_E_4_) [5] and the gelled lamellar phase L_α_ of the system H_2_O–2C_12_DAB–12-HOA [6]. The cell surely is the most prominent example. Proteins form filaments, which provide mechanical stability of the cell and coexist independently with the lipid bilayer of phospholipids, i.e., the building block of the cell membrane [3]. Warriner et al. introduced lamellar biological hydrogels that consist of the lipid dimyristoyl phosphatidyl choline (DMPC), the co-surfactant pentanol and tiny amounts of poly (ethylene glycol)-derived polymer lipids (PEG-lipids) [4]. The domains of the lamellar membranes built by DMPC and pentanol are randomly orientated and provide the gel properties, while the PEG-lipids are attached to these membranes, but can diffuse freely within the lamellar bilayers, which are separated by water. These PEG-lipids stabilize defects occurring in high curvature regions and lead to highly flexible membranes with large distances from each other caused by long-range repulsive forces of the PEG-lipids. Xu et al. studied the gelled lamellar phase L_α_ of the system H_2_O–*n*-decane/12-HOA–C_10_E_4_ with a focus on the question whether the formation of the gel network and of the lamellar phase formation is simultaneous, but independent [5]. Koitani et al. tried to answer the same question by studying the lamellar phase L_α_ of the system H_2_O–2C_12_DAB–12-HOA [6].

Introduced by Laibinis et al. for alkanethiols and alkane carboxylic acids on gold and alumina, respectively, the simultaneous (but independent) formation of two coexisting structures is called orthogonal self-assembly [7]. However, the term orthogonal self-assembly is not restricted to surface chemistry. Orthogonal self-assembled structures have been studied in bulk systems as well [8,9,10,11,12]. The cell, once again, is the most prominent example of an orthogonal self-assembled system, since proteins self-assemble to fibers forming the cytoskeleton, while phospholipids self-assemble to membranes [3]. The expression orthogonal self-assembly was first used for gelled complex fluids by the group of van Esch [13]. They reported on surfactant micelles, worm-like micelles, liposomes and phospholipids entrapped in self-assembled fibrillary networks of low molecular weight gelators (LMWG) [13,14,15,16,17]. Laupheimer studied the system H_2_O–*n*-decane/12-HOA–C_10_E_4_ and proved that the gelled bicontinuous microemulsion is an orthogonal self-assembled system in which the formation of the nanostructured microemulsion is nearly independent on the formation of the gel network [18,19,20]. In the special case of the gelled L_α_ phase of the very same system, the question whether it is a truly orthogonal self-assembled system could not be answered satisfactorily. On the one hand, phase behaviour and rheological studies supported the idea of an orthogonal self-assembled gelled lamellar L_α_ phase. On the other hand, freeze fracture electron microscopy revealed that the gel network and the lamellar L_α_ phase influence each other: The presence of the gel network leads to a higher order of the L_α_ phase while the presence of the L_α_ phase results in gel fibers which are not twisted anymore [5]. Moreover, Laupheimer et al. showed in a previous SANS study of the system H_2_O–*n*-decane/12-HOA–C_10_E_4_ that 12-HOA is only partly involved in the gel formation, since it also acts as co-surfactant, which results in a shift of the phase boundaries to lower temperatures [20]. Koitani et al. studied the gelled lamellar phase L_α_ of the system H_2_O–didodecyldimethylammonium bromide (2C_12_DAB)–12-HOA at surfactant mass fractions γ_a_ = 0.10 and γ_a_ = 0.20 in order to answer the question of orthogonal self-assembly [6]. However, they also found that 12-HOA seems to have two roles in surfactant-containing systems, namely as gelator and as co-surfactant. The slightly surface active 12-HOA molecules are partly incorporated in the surfactant bilayers of the system H_2_O–2C_12_DAB–12-HOA and are thus not available for the gel formation which leads to a weaker gel compared to the binary gel *n*-decane–12-HOA. Additionally, the incorporation of 12-HOA in the bilayers caused a slightly larger interlayer spacing compared to the non-gelled L_α_ phase. In order to elucidate the role of 12-HOA in surfactant-containing systems and its ability of gelling lyotropic liquid crystals of different phase structures, we chose the binary system H_2_O–heptaethylene glycol monododecyl ether (C_12_E_7_) instead of the ternary system H_2_O–*n*-decane–C_10_E_4_ or the system H_2_O–2C_12_DAB, for the following reasons. Firstly, studying the system H_2_O–C_12_E_7_ ensures easy handling and a broader access to gelled lyotropic liquid crystals as it forms three lyotropic liquid crystalline phases, namely the hexagonal phase H_1_, the bicontinuous cubic phase V_1_ and the lamellar phase L_α_ in specific surfactant concentration ranges and with melting points at moderate temperatures (*T* ≈ 50 °C) [21]. Secondly, it allows us to prove the concept of gelling lyotropic liquid crystals of binary water–surfactant systems with the organogelator 12-HOA at much higher surfactant concentrations as compared to the binary system H_2_O–2C_12_DAB. We first investigated the influence of the 12-HOA concentration on the phase boundaries of the system H_2_O–C_12_E_7_ by means of visual observation in water basins and determined qualitatively whether a gelled lyotropic liquid crystalline phase was formed. ^2^H NMR spectroscopy complemented the visual phase studies, since it is a well-known and powerful tool to detect phase transitions of lyotropic liquid crystalline phases to an isotropic phase and the coexistence of two phases, respectively [22,23,24,25,26,27,28,29,30]. In addition, we investigated the rheological properties of the lyotropic liquid crystalline phases in the presence of 12-HOA in order to identify gelled lyotropic liquid crystalline phases.

## 2. Results and Discussion

### 2.1. Visual Phase Studies

**Figure 1:**
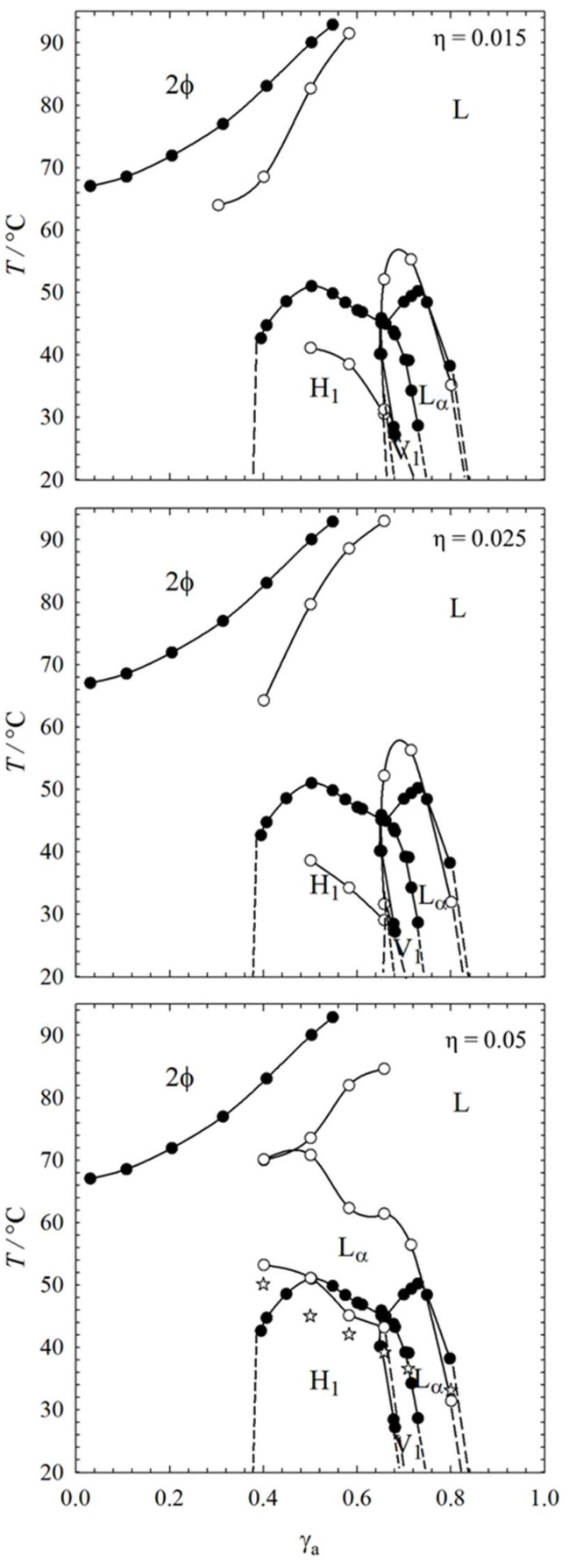
*T*–γ_a_ phase diagrams of the binary system H_2_O–C_12_E_7_ (black circles) and of the systems H_2_O–C_12_E_7_–12-HOA (open circles) with 12-hydroxyoctadecanoic acid (12-HOA) mass fractions η = 0.015, 0.025, and 0.05 (from top to bottom). The phase transition temperatures were determined by visual observations in water basins. The stars in the phase diagram of the system H_2_O–C_12_E_7_–12-HOA with η = 0.05 indicate the approximate sol-gel transition temperatures. Gelation could not be observed for H_2_O–C_12_E_7_–12-HOA with 12-HOA mass fractions of η = 0.015 and η = 0.025. The error is within the size of the symbols.

The visual phase studies allowed us to determine the occurrence of lyotropic liquid crystals via optical birefringence. In addition, they helped identifying transition temperatures, namely the melting points of the lyotropic liquid crystals and the sol-gel transition of the gel network. Therefore, we were able to measure the influence of 12-HOA on the phase boundaries of the lyotropic liquid crystalline phases of the system H_2_O–C_12_E_7_. As already mentioned, the system H_2_O–C_12_E_7_ forms three liquid crystalline phases as a function of the surfactant concentration, namely the hexagonal phase H_1_, the bicontinuous cubic phase V_1_ and the lamellar phase L_α_. The visually determined *T*-γ_a_ phase diagram of the binary system H_2_O–C_12_E_7_, as well as those of the systems in the presence of 12-HOA, i.e., H_2_O–C_12_E_7_–12-HOA with 12-HOA mass fractions η = 0.015, 0.025, 0.05, are shown in Figure 1.

The black circles refer to the binary system H_2_O–C_12_E_7_ and we note that the phase boundaries are in good agreement with literature data [21]. The highly viscous and transparent hexagonal H_1_ phase occurs at a surfactant concentration range of γ_a_ ≈ 0.40–0.68 and melts at temperatures *T* ≈ 50 °C. At higher surfactant concentrations, in a range of γ_a_ ≈ 0.72–0.85, the lamellar L_α_ phase is formed. The L_α_ phase melts at about the same temperatures as the hexagonal H_1_ phase, but is less viscous and appears slightly turbid. The highly viscous, transparent bicontinuous cubic phase V_1_ occurs between the hexagonal phase H_1_ and the lamellar phase L_α_ in a surfactant concentration range γ_α_ ≈ 0.68–0.72 and melts at temperatures *T* ≈ 46 °C. In contrast to the anisotropic H_1_ and L_α_ phases, the bicontinuous cubic phase V_1_ is characterized by optical isotropy. The V_1_ phase will not be discussed further as it is difficult to identify, especially in the presence of 12-HOA. The upper miscibility gap (2φ), which is typical for oligo (ethylene oxide) alkyl ethers, was detected at higher temperatures for surfactant concentrations up to γ_a_ = 0.50. 

The open circles in Figure 1 represent the phase boundaries of the system H_2_O–C_12_E_7_ at three different 12-HOA concentrations. Comparing the binary system with the systems in the presence of 12-HOA, it is obvious that 12-HOA has an enormous influence on the phase boundaries of the liquid crystalline phases. For a 12-HOA mass fraction of η = 0.015, the melting point of the lamellar phase L_α_ is increased by Δ*T ≈* 5 K, whereas at the same time the melting point of the hexagonal phase H_1_ is decreased by the same value. The upper miscibility gap is also shifted towards lower temperatures in the presence of 12-HOA. The influence of 12-HOA on the upper miscibility gap at surfactant concentrations γ_a_ < 0.3 could not be determined via visual observations, because 12-HOA precipitated at these surfactant concentrations. After addition of 12-HOA (η = 0.015) the appearance and viscosity of the lamellar phase L_α_ did not change. It maintained its slight turbidity and also its flow ability, i.e., no gelled lamellar L_α_ phase was formed. On the contrary, the initially transparent hexagonal phase H_1_ became turbid after addition of 12-HOA (η = 0.015), but whether a gel was formed, could not be detected by visual observation, because the viscosity of the hexagonal phase H_1_ is high a priori. Moreover, the turbidity of the hexagonal phase complicated the visual observation, especially at low temperatures. For a 12-HOA concentration of η = 0.025 we observed the same trend, i.e., a stabilized lamellar L_α_ phase and a destabilized hexagonal H_1_ phase, but a bit more pronounced as for η = 0.015. Again, even after increasing the 12-HOA concentration to η = 0.025, the appearance of the lamellar phase L_α_ did not change, i.e., still no gelled lamellar phase L_α_ was formed. In the case of the hexagonal phase H_1_, the effect of 12-HOA is also slightly more pronounced for η = 0.025 than for η = 0.015. The hexagonal H_1_ phase is marginally less temperature stable for η = 0.025, but again its appearance changes from transparent to turbid. For the highest 12-HOA concentration, i.e., η = 0.05, the lamellar phase L_α_ is extended to higher temperatures and lower surfactant concentrations γ_a_, i.e., it additionally occurs in the range of γ_a_ = 0.40–0.60 at temperatures *T* = 50–70 °C. Unlike for η = 0.015 and 0.025, gelled L_α_ phases were obtained for η = 0.05 in a surfactant concentration range of γ_a_ = 0.72–0.85. In addition, gelled isotropic solutions were formed at lower concentrations of γ_a_ = 0.40–0.60, since the hexagonal H_1_ phase did not form anymore in the presence of 12-HOA at η = 0.05. In Figure 1, bottom, the approximate sol-gel transition temperature line is added to the phase diagram of the system H_2_O–C_12_E_7_–12-HOA (η = 0.05) and it can be seen that the sol-gel transition temperatures decrease with increasing surfactant concentration γ_a_. 

Based on these results it is clear that 12-HOA has two roles in the system H_2_O–C_12_E_7_–12-HOA. On the one hand, it acts as co-surfactant, which results in the stabilization of the lamellar phase L_α_ and at the same time in the destabilization of the hexagonal phase H_1_. This effect can be explained by Israelachvili’s packing parameter, which is the ratio of hydrophobic area to the effective hydrophilic head group area [31]. By adding 12-HOA to the system H_2_O–C_12_E_7_, a C_18_ chain is introduced which increases the volume of the hydrophobic chains. As a consequence, the packing parameter is increased, and the surfactant layer has a lower curvature. This stabilizes the lamellar phase L_α_, and simultaneously destabilizes the hexagonal phase H_1_ [31,32,33]. On the other hand, it seems that at sufficient high gelator concentrations, i.e., for η > 0.025–0.05, the bilayers of the lamellar phase are saturated by 12-HOA and 12-HOA starts to act as gelator. The sol-gel transition temperature of the system H_2_O–C_12_E_7_–12-HOA (η = 0.05) decreases with increasing surfactant concentration γ_a_ which can be explained by an increasing surfactant layer at higher surfactant concentrations γ_a_. The higher the surfactant concentrations γ_a_, the more likely 12-HOA is incorporated in the surfactant layer instead of forming the gelator network, which leads to weaker gels with lower sol-gel transition temperatures *T*_sol-gel_. The system H_2_O–C_12_E_7_ thus is a better solvent for 12-HOA at high surfactant concentrations γ_a_ than at low ones. This concentration dependence is already evident at the lowest 12-HOA concentration η = 0.015. While the appearance of the lamellar L_α_ phase at high surfactant mass fractions does not change in the presence of 12-HOA at η = 0.015, the otherwise clear hexagonal H_1_ phase becomes turbid, which may indicate the start of a gel formation. Therefore, we focused our study on the system H_2_O–C_12_E_7_–12-HOA at η = 0.015, in order to answer the question whether 12-HOA acts as both gelator and co-surfactant already at the lowest 12-HOA mass fraction. 

### 2.2. ^2^H NMR

**Figure A1:**
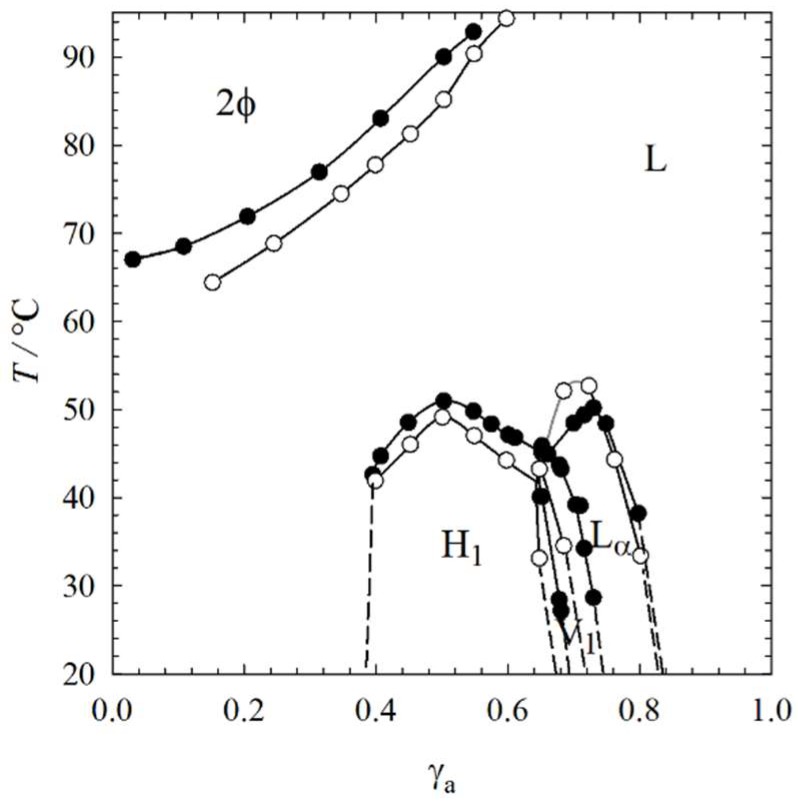
*T*–γ_a_ phase diagrams of the binary systems H_2_O–C_12_E_7_ (black circles) and D_2_O–C_12_E_7_ (open circles). The phase transition temperatures were determined by visual observation in water basins.

**Figure A2:**
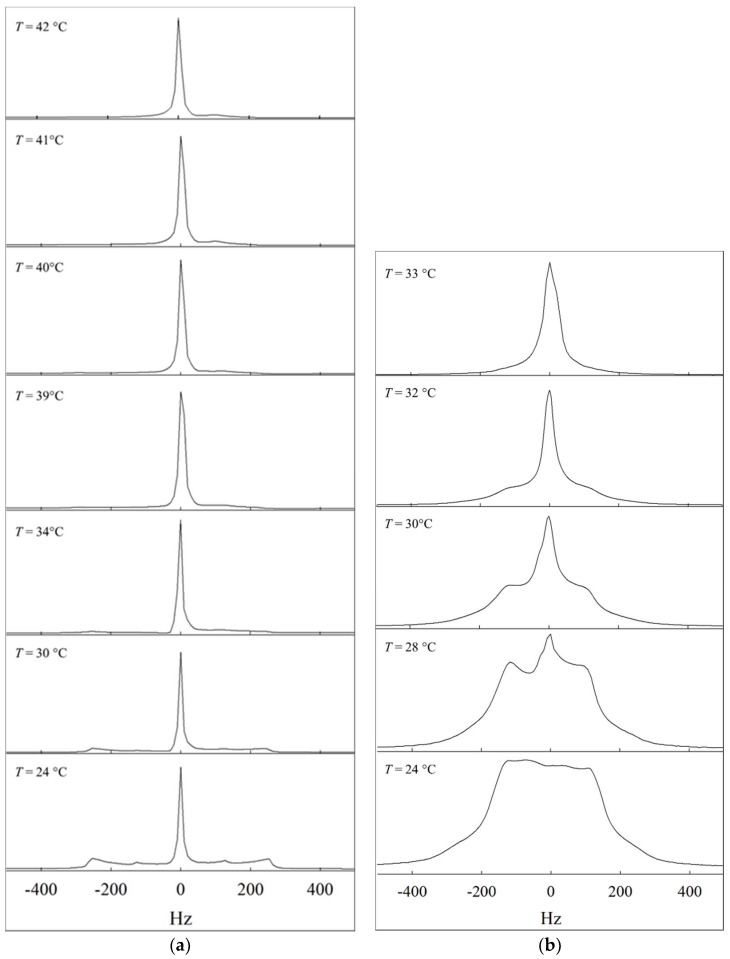
(**a**) Temperature dependent ^2^H NMR spectra of the system D_2_O–C_12_E_7_ recorded at γ_a_ = 0.37. (**b**) Temperature dependent ^2^H NMR spectra of the system D_2_O–C_12_E_7_–12-HOA recorded at γ_a_ = 0.36 and at η = 0.015. The asymmetry seen in the spectra may have several reasons. One is the local anisotropy, which causes an anisotropy of the magnetic susceptibility and therefore an orientation dependent shift in addition to the orientation dependent quadrupolar splitting. Therefore, the isotropic peak is not exactly in the center of the two maxima (which correspond to domains aligned with the phase axis perpendicular to the magnetic field), and the maxima are not exactly in the middle of the two outer shoulders (which correspond to domains aligned with the phase axis parallel to the magnetic field). Additional asymmetries arise from shim and phase errors.

We performed ^2^H NMR measurements in order to complement the visual phase studies of the system H_2_O–C_12_E_7_–12-HOA at η = 0.015 as the determination of the phase boundary of the hexagonal phase H_1_ at surfactant concentrations γ_a_ ≈ 0.40 is difficult by means of visual observation, in particular, in the presence of 12-HOA which makes the sample turbid. For this purpose, H_2_O needed to be replaced by D_2_O, which, in turn, requires a phase study of D_2_O–C_12_E_7_. The phase diagram of D_2_O–C_12_E_7_ is available in the Appendix A (Figure A1). It was found that the lyotropic liquid crystalline phases were shifted to slightly smaller surfactant mass fractions γ_a_. Moreover, the phase boundary of the hexagonal phase H_1_ was shifted by Δ*T* = 1–2 °C to lower values, whereas the phase boundary of the lamellar phase L_α_ was shifted to higher temperatures by Δ*T* = 2–3 °C. The miscibility gap at low surfactant concentrations and at high temperatures was shifted to lower temperatures by Δ*T* = 2–3 °C, i.e., it seems that the surfactant is more hydrophobic in the presence of D_2_O [29]. The temperature shifts caused by replacing H_2_O by D_2_O are the same for the systems without and with 12-HOA. We performed temperature dependent ^2^H NMR measurements at γ_a_ = 0.37 and 0.38 for the system D_2_O–C_12_E_7_ and at γ_a_ = 0.36 and 0.38 for the system D_2_O–C_12_E_7_–12-HOA (η = 0.015). In the following, we will discuss the ^2^H NMR spectra of the systems D_2_O–C_12_E_7_ and D_2_O–C_12_E_7_–12-HOA (η = 0.015) at γ_a_ = 0.38. The spectra of the systems D_2_O–C_12_E_7_ at γ_a_ = 0.37 and D_2_O–C_12_E_7_–12-HOA (η = 0.015) at γ_a_ = 0.36 are shown in the Appendix A (Figure A2). 

In the anisotropic hexagonal phase H_1_, the quadrupolar ^2^H nucleus with a spin quantum number *I* = 1 shows a splitting which depends on the local orientation order of the D_2_O molecules, as well as on the orientation of the director of the hexagonal phase with respect to the magnetic field. The D_2_O molecules are on average slightly aligned, since they interact with the aligned surfactant molecules resulting in a non-zero residual quadrupole coupling. Hexagonal phases H_1_ are not macroscopically aligned by the magnetic field, because of their high viscosity, i.e., domains with all possible orientations of the hexagonal axis are present in the sample. The superposition of the spectral contributions from all domains leads to a characteristic line shape, called the Pake powder pattern [34]. On the contrary, isotropic phases are indicated by a single peak, since the temporal average over the random orientations of the molecules, which have no preferred orientation with respect to the magnetic field eliminates the quadrupole coupling. 

**Figure 2:**
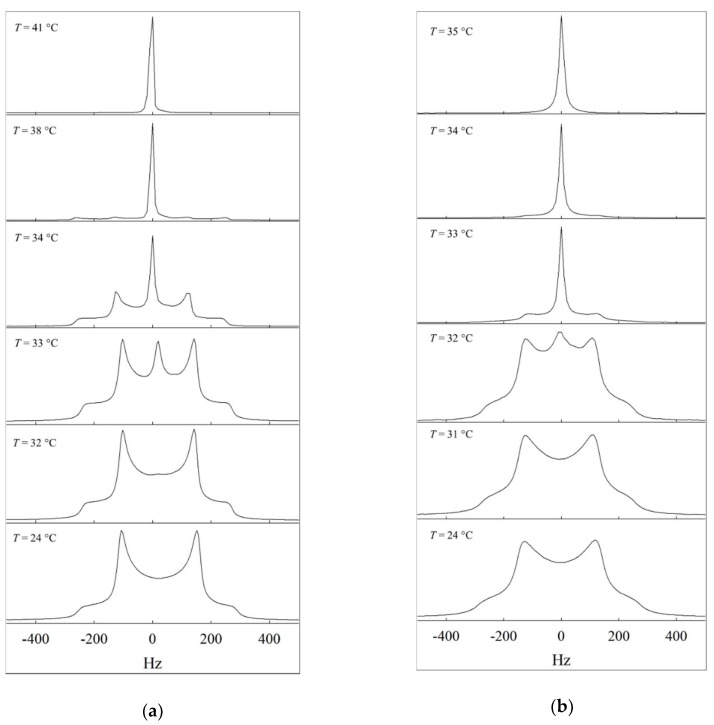
(**a**) Temperature dependent ^2^H NMR spectra of the system D_2_O–C_12_E_7_, recorded at γ_a_ = 0.38. (**b**) Temperature dependent ^2^H NMR spectra of the system D_2_O–C_12_E_7_–12-HOA, recorded at γ_a_ = 0.38 and η = 0.015.

In Figure 2a, the ^2^H NMR spectra of the system D_2_O–C_12_E_7_ at γ_a_ = 0.38 as a function of temperature are shown. The bottom two spectra show the Pake powder pattern with two inner peaks and two outer shoulders, corresponding to domains with the hexagonal axis perpendicular and parallel to the magnetic field, respectively. The shape of the Pake pattern represents the isotropic distribution of domains in a macroscopically unaligned sample. As we studied the system D_2_O–C_12_E_7_ by means of visual observation, and since the general phase behavior is known in literature, the splitting can be assigned to the hexagonal phase H_1_. The spectrum at *T* = 33 °C shows an additional peak that can be assigned to an isotropic phase that coexists with the hexagonal phase. The two phase region can be detected up to *T* = 40 °C, though the peaks that indicate the hexagonal phase are at minimum intensity. At *T* = 41 °C the single peak corresponds to an isotropic phase, therefore this temperature is set as phase transition temperature from the hexagonal phase H_1_ to the isotropic phase. The ^2^H NMR spectra of the system D_2_O–C_12_E_7_ at γ_a_ = 0.37 shown in the Appendix A indicate that the system consists of an isotropic and a hexagonal phase H_1_ from room temperature up to *T* = 42 °C. 

The respective temperature dependent ^2^H NMR spectra of the system D_2_O–C_12_E_7_–12-HOA (η = 0.015) at γ_a_ = 0.38 are shown in Figure 2b. As opposed to the spectra of the system D_2_O–C_12_E_7_, the line shape is less sharp in the presence of 12-HOA, though the general phase behavior is maintained in presence of 12-HOA, but shifted to lower temperatures by Δ*T* = 8 °C, which is in accordance with the visual phase studies. At *T* = 25 °C up to *T* = 31 °C again the Pake pattern can be observed and can also be assigned to the hexagonal phase H_1_. At *T* > 32 °C an additional peak occurs, which can be assigned to the isotropic phase. At *T* = 34 °C, the Pake spectrum is still visible, indicating that the system is still in the two phase region. The phase transition temperature from the hexagonal phase H_1_ to the isotropic phase was set to *T* = 35 °C—since a single peak, indicative of a pure isotropic phase, was recorded. In the Appendix A the spectra of the 12-HOA containing sample at γ_a_ = 0.36 are shown. 

**Figure 3:**
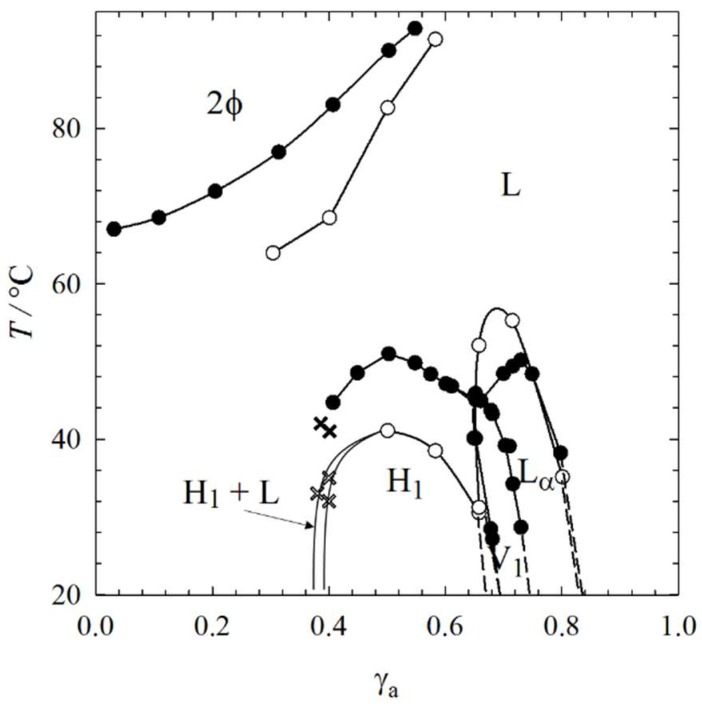
*T–*γ_a_ phase diagram of the binary system H_2_O–C_12_E_7_ (black symbols) and of the system H_2_O–C_12_E_7_–12-HOA (open symbols) at η = 0.015. The phase transition temperatures were determined by visual observation in water basins (circles) and by ^2^H NMR (crosses). For the sake of clarity, the two-phase region is not drawn for the system H_2_O–C_12_E_7_.

In Figure 3, the phase transitions determined by ^2^H NMR are added to the phase diagram of the system H_2_O–C_12_E_7_–12-HOA (η = 0.015) determined by visual observation in water basins (see Figure 1, top). We added Δ*T* = +1 to the phase transitions determined by ^2^H NMR before we added them to the phase diagram, since D_2_O influences the phase boundaries in that way (see Figure A1). The results of visual observations and ^2^H NMR are in good agreement and complement each other. We were able to detect the phase boundaries of the hexagonal phase H_1_ at γ_a_ < 0.40 in the presence of 12-HOA, which was difficult by means of visual observations, due to turbidity caused by 12-HOA. Moreover, the ^2^H NMR measurements enabled the detection of the two-phase region of isotropic and hexagonal phase at γ_a_ = 0.38–0.40. To summarize, ^2^H NMR measurements confirm the phase behavior and the role of 12-HOA as co-surfactant determined by visual observations. 

### 2.3. Rheometry 

**Figure 4:**
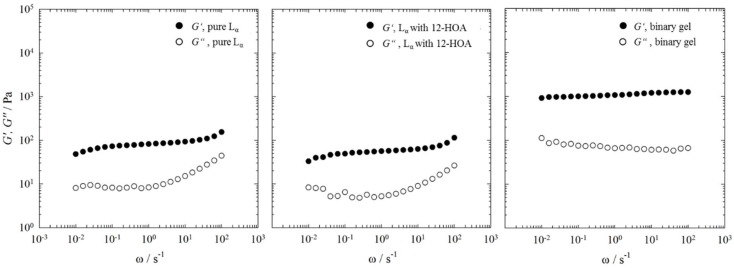
Storage modulus *G’* and loss modulus *G’’* (filled and open circles) of the pure lamellar phase L_α_ at γ_a_ = 0.76 (**left**), of the lamellar phase L_α_ at γ_a_ = 0.76 in presence of 12-HOA at η = 0.015 (**middle**), and of the binary gel n-decane–12-HOA at η = 0.015 (**right**) determined by frequency sweeps at T = 22 °C and a strain amplitude of γ = 1%.

**Figure 5:**
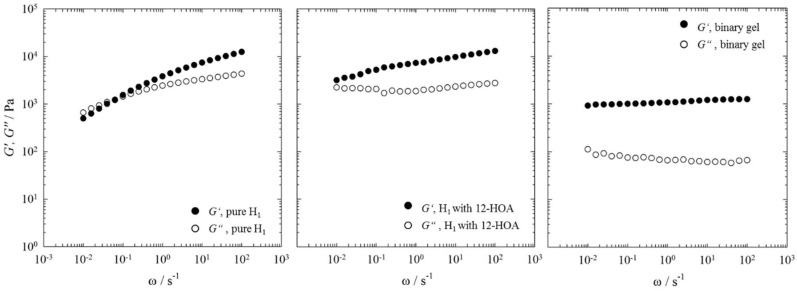
Storage modulus *G’* and loss modulus *G’’* (filled and open circles) of the pure hexagonal phase H_1_ at γ_a_ = 0.50 (**left**), of the hexagonal phase H_1_ at γ_a_ = 0.50 in presence of 12-HOA at η = 0.015 (**middle**), and of the binary gel n-decane–12-HOA at η = 0.015 (**right**) determined by frequency sweeps at *T* = 22 °C and a strain amplitude of γ = 1%.

Looking at the phase diagrams and the NMR spectra one can conclude that 12-HOA acts as co-surfactant up to η = 0.015. Somewhere between a 12-HOA concentration of η = 0.025 and η = 0.05 its second role of a gelator becomes obvious. However, at the lowest 12-HOA concentration of η = 0.015, the hexagonal H_1_ phase went turbid as discussed in Section 2.1, which could indicate the beginning of a gel formation. In other words, it can well be that 12-HOA acts already as both co-surfactant and gelator at η = 0.015. In order to determine whether or not the hexagonal phase H_1_ was gelled at a 12-HOA concentration of η = 0.015, we performed frequency sweeps by oscillation shear rheometry and compared the results with the pure hexagonal phase H_1_ at γ_a_ = 0.50 and with the binary gel *n*-decane–12-HOA at η = 0.015. For comparison, we also studied the lamellar L_α_ phase at γ_a_ = 0.76 under the same conditions. The results of the frequency sweeps are shown in Figure 4 for the lamellar phase L_α_ and in Figure 5 for the hexagonal phase H_1_, respectively.

For all systems, except for the pure hexagonal phase H_1_ (Figure 5, left) the storage modulus *G’* is higher than the loss modulus *G’’* over the whole frequency range investigated, i.e., the binary gel *n*-decane–12-HOA, the pure lamellar phase L_α_, as well as both the lamellar phase L_α_ and the hexagonal phase H_1_ in presence of 12-HOA at η = 0.015 show solid like behavior. In case of the pure hexagonal phase H_1_ a crossover point between *G’* and *G*’’ is observed at frequencies of ω ≈ 0.1 s^−1^, which indicates a change from solid like, elastic behavior (*G’* > *G’’*) to liquid like, viscous behavior (*G’* < *G’’*) [35]. Moreover, *G’* of the pure hexagonal phase H_1_ changes with the frequency. Characteristic for gels and soft solids is the frequency independence of *G’* as can be seen in the frequency sweep of the binary gel *n*-decane–12-HOA [36,37,38,39]. The frequency sweeps of the pure lamellar phase L_α_, as well as of the lamellar phase L_α_ and the hexagonal phase H_1_ in presence of 12-HOA show a slight frequency dependence of *G*’. Comparing the absolute values of *G’* and *G’’*, one sees that the pure hexagonal phase H_1_ is much more viscous than the pure lamellar phase L_α_. This results from the densely packed cylinders compared to the parallel orientated lamellar bilayers, which can easily slip against each other [40]. Moreover, the absolute *G’* and *G’’* values of the pure lamellar phase L_α_ are lower than those of the binary gel and do not change in the presence of 12-HOA. This shows that no gel was formed in case of the lamellar phase L_α_. In the frequency sweep of the hexagonal phase H_1_ in presence of 12-HOA no crossover between *G’* and *G’’* can be seen anymore, since the slope of *G’* is lower, i.e., the solid like behavior predominates in the whole frequency range and indicates that a gel was indeed formed. However, as the storage modulus *G’* is still frequency dependent, a gel weaker (but stiffer) than the binary gel was formed. Note that the absolute values of *G’* and *G’’* cannot be used as measure for gel formation as they are a priori high for the pure hexagonal phase H_1_. In conclusion, one can say that for the lowest 12-HOA mass fraction of η = 0.015, 12-HOA is only co-surfactant in case of the lamellar L_α_ phase, but acts as both co-surfactant and gelator in case of the hexagonal phase. Again, it becomes obvious that 12-HOA is more likely incorporated in the surfactant layer of the system H_2_O–C_12_E_7_ at higher surfactant mass fractions than at low ones—since at higher surfactant mass fractions most of the 12-HOA molecules are dissolved in the surfactant layer, no gel can be formed. 

## 3. Conclusions

Both visual observation and ^2^H NMR clearly show that 12-HOA stabilizes the lamellar phase and destabilizes the hexagonal phase of the binary system H_2_O–C_12_E_7_ (heptaethylene glycol monododecyl ether). The strong influence of 12-HOA on the phase behavior is clear evidence that the organogelator acts as a co-surfactant. With increasing mass fraction η of 12-HOA the effect is more pronounced (at the highest gelator mass fraction investigated, namely η = 0.05, the hexagonal phase is completely absent), but the gelation capacity is increased, too. At η = 0.05, a sol-gel transition can be observed by visual inspection. The sol-gel transition temperature decreases with increasing surfactant concentration, which proves a decreasing gelation capacity, due to an increasing incorporation of 12-HOA in the surfactant layer. Note that the total gelator mass fraction is kept constant, while the amount of surfactant thus the surfactant layer increases. 

The ^2^H NMR measurements show broadened features of the Pake powder pattern of the hexagonal phase, which indicates an increased viscosity, even at the lowest gelator mass fraction of η = 0.015. Dynamic rheometry proves that a weak gel is formed at this low gelator concentration in the case of the hexagonal phase (i.e., at low surfactant concentrations), whereas no gel is formed in the case of the lamellar phase (i.e., at high surfactant concentrations). This is in excellent agreement with the decreasing gelation capacity deduced from decrease of the sol-gel transition temperature as a function of surfactant concentration.

Our investigations clearly show that 12-HOA is not a suitable gelator for binary systems consisting of H_2_O and an alkyl polyglycol ether (C_i_E_j_), since its gelation capacity is hampered by its competing role as co-surfactant. The search for a suitable gelator for H_2_O–C_i_E_j_ systems that solely acts as gelator is continued; tests of other organogelators, as well as hydrogelators, are in progress. Once a suitable gelator is found, we will investigate (a) if the lyotropic liquid crystalline phases serve as template for the gelator network leading to well aligned gelator fibers and (b) if the gel network influences the structure of the lyotropic liquid crystalline phases. For this purpose, the sol-gel transition line has to be adjusted via the gelator concentration, such that (a) the sol-gel transition temperature *T*_sol-gel_ of the gelator is lower than the melting point of the lyotropic liquid crystalline phases, and (b) the sol-gel transition temperature *T*_sol-gel_ of the gelator is higher than the melting point of the lyotropic liquid crystalline phases. First experimental evidence that the chronology—that is, whether gelation or liquid crystal phase formation occurs first—may indeed play a role is the work published by Kato et al. They studied gelled thermotropic liquid crystals (thermotropic LC), focusing on the influence of the chronology on the resulting structure [41].

## 4. Materials and Methods

### 4.1. Materials and Sample Preparation

We purchased heptaethylene glycol monododecyl ether (C_12_E_7_) from Sigma Aldrich (≥98%) (St. Louis, MO, USA), Nikkol (Tokyo, Japan) and TCI (Tokyo, Japan), 12-hydroxyoctadecanoic acid (12-HOA) from Alfa Aesar (95%) (Karlsruhe, Germany), and D_2_O from euriso-top (99.9 atom % D). Note that Alfa Aesar does not specify whether 12-HOA is a racemate or a pure enantiomer. We thus measured the optical rotation angle of 12-HOA in methanol and found out that the substance is the (*R*)-enantiomer. In a previous study we proved that 12-HOA provided by Sigma Aldrich is also (*R*)-12-HOA. [42] All chemicals were used without further purification. 

The composition of the systems is defined by the surfactant mass fraction,

(1) $$\gamma_{a}=\;\frac{m_{C_{12}E_{7}}}{m_{C_{12}E_{7}}{+\;m}_{H_{2}O}},\;$$

and the 12-HOA mass fraction,

(2) $$\eta \;=\;\frac{m_{12\text{-}\mathrm{HOA}}}{m_{C_{12}E_{7}}{+\;m}_{H_{2}O}{+\;m}_{12\text{-}\mathrm{HOA}}\;}.\;$$

We used η = 0.015, 0.025, 0.05 as 12-HOA mass fractions. The samples were weighed in glass tubes, equipped with a stirring bar and sealed with plugs. The samples had to be heated in a water bath up to *T* = 85 °C as 12-HOA has to be molten and they were then stirred at this temperature for at least 5 min in order to ensure homogenous mixing. Subsequently, the samples were put into an ice bath for gelation for 5 min. Gelation was indicated by a change of turbidity in the case of the otherwise clear hexagonal phase and a change in viscosity in the case of the lamellar phase L_α_. It is important to note that changes in turbidity and viscosity are no proofs of gelation per se. However, in the present case they can be used to identify gelation, since there are no other (phase) transitions, which could explain these changes. The samples measured by ^2^H NMR were prepared with D_2_O instead of H_2_O, but the surfactant mass fraction, as well as the 12-HOA mass fraction were calculated in the same way. 

### 4.2. Visual Phase Studies

The phase boundaries of the binary system H_2_O–C_12_E_7_ and of the systems H_2_O–C_12_E_7–_12-HOA with η = 0.015, 0.025, 0.05 were determined by visual observation in water basins equipped with a thermostat (Thermo Scientific DC30, Waltham, MA, USA). The phase boundaries of the anisotropic lyotropic LC phases to the transparent, isotropic phase were detected with the help of a lamp behind the water basin and two crossed polarizers, one behind and one in front of the sample. The lyotropic LC phases were distinguished due to anisotropy, viscosity and turbidity. The hexagonal phase H_1_ is transparent and highly viscous, whereas the lamellar phase L_α_ is slightly turbid and less viscous. Accordingly, the turbid upper miscibility gap at higher temperature was identified. The approximate sol-gel transition temperature was determined by fading turbidity in the case of the hexagonal phase H_1_ and by a change in viscosity in the case of the lamellar phase L_α_. 

### 4.3. 2H NMR

^2^H NMR measurements were carried out with a Tecmag Apollo 300 MHz spectrometer (Erfurt, Germany) at a ^2^H resonance frequency of 46.02 MHz with a pulse width of 6.4 µs and a relaxation delay time of 2 s. The samples were filled in 4 cm glass tubes with a diameter of 5 mm. The tubes, equipped with a Teflon spacer and sealed with a Teflon plug and parafilm, were put in a goniometer probe such that its axis was perpendicular to the magnetic field. By applying a quadrupole echo pulse sequence, temperature-dependent ^2^H-NMR spectra of each sample were recorded. The sample temperature in the goniometer probe was set according to the temperature calibration of a reference probe, which was calibrated by a known phase transition of the system that was determined by visual observation before. The target temperatures were reached after a 30 min heating ramp followed by an additional 30 min equilibration time. 64 scans were averaged before Fourier transformation was applied. 

### 4.4. Rheometry

The measurements were carried out with a Physica MCR 501 rheometer from Anton Paar (Ostfildern, Germany). A cone-plate geometry was used with an upper moving cone of 2.5 cm diameter and a cone angle of 1°. The samples were transferred to the plate with a spatula. After the upper cone was lowered to the measuring position (gap width *z* = 1 mm), the samples were kept at *T* = 22 °C for 30 min to reach equilibrium. Then, frequency (ω) sweeps were performed with a constant strain amplitude γ = 1% and constant temperature *T* = 22 °C of the lower plate. The frequency ω = 0.01 s^−1^ is the lower limit of our rheometer. The temperature was set with a precision of ±0.1 K by a Peltier element. The strain amplitude γ was set such that it was in the linear viscoelastic (LVE) region determined through prior oscillating stress sweeps. The storage modulus *G’* and the loss modulus *G’’* were determined in the frequency sweeps. The pure hexagonal H_1_ phase at a surfactant mass fraction γ_a_ = 0.50 and the pure lamellar L_α_ phase at γ_a_ = 0.76 as well as both lyotropic liquid crystalline phases in the presence of 12-HOA with a gelator mass fraction η = 0.015 were measured. For comparison, the rheological behaviour of the binary gel *n*-decane–12-HOA at η = 0.015 was investigated.
